# Comparing Ovarian Clear Cell Carcinoma and High-Grade Serous Carcinoma Based on the SEER Database and Analyzing the Significantly Mutated Genes

**DOI:** 10.2174/0115680096370568250806153217

**Published:** 2025-08-28

**Authors:** Xuzhi Liang, Ying Yang, Shiyu Zhang, Haijing He, Yuqi Wen, Jiangtao Fan

**Affiliations:** 1 Department of Gynecology, Guangxi Medical University First Affiliated Hospital, Nanning, Guangxi, China

**Keywords:** Ovarian clear cell carcinoma, high-grade serous carcinoma, prognosis, whole exome sequencing, significantly mutated genes, tumor mutation burden, target drugs

## Abstract

**Introduction:**

Ovarian clear cell carcinoma (OCCC) accounts for about 5% of all epithelial ovarian cancers. Currently, its treatment mainly refers to high-grade serous carcinoma (HGSC). This study aimed to explore differences in clinical characteristics between OCCC and HGSC and studied the reasons for the differences.

**Methods:**

The data of OCCC and HGSC cases were obtained from the SEER database. Univariate and multivariate Cox regression analyses were used to explore the prognostic factors. Next, whole exome sequencing (WES) was performed on 15 clinically selected OCCC cases and 16 HGSC cases to identify significantly mutated genes (SMGs). Further analysis included calculating tumor mutation burden (TMB) and predicting potential target drugs based on the identified mutations.

**Results:**

3493 OCCC and 10266 HGSC patients from the SEER database were included in the study. Survival analysis showed that the overall survival (OS) of stage I-II OCCC was better than that of stage I-II HGSC, while the OS of stage III-IV OCCC was worse than that of stage III-IV HGSC. Further subgroup analysis showed that for the OCCC group, age ≥ 60 years, bilateral tumor distribution, tumor size ≥ 87mm, and stage III-IV were independent risk factors for OS. For HGSC patients, tumor size ≥ 87mm was an independent protective factor for OS. WES results suggested that among the top 20 SMGs of OCCC in stage III-IV patients, DNAH2, LAMA5, MUC19, NOTCH1, PCLO, SYNE2, TACC2, and ZNF469 were 8 specific SMGs that distinguish III-IV OCCC from III-IV HGSC. In addition, the stage I-II OCCC group had the highest TMB, and the lowest was the stage III-IV OCCC.

**Discussion:**

Our findings challenge the conventional uniform therapeutic approach for ovarian carcinomas by revealing stage-dependent SMGs between OCCC and HGSC. However, limitations such as the retrospective SEER analysis, small WES cohort, and population-specific driver gene variations require cautious interpretation of the findings.

**Conclusion:**

The independent prognostic factors identified in this study provide a theoretical basis for individualized prognosis judgment in OCCC and HGSC. The SMGs and TMB levels may serve as valuable indicators for prognosis and evaluating targeted therapy or immunotherapy efficacy. Druggable genes such as NOTCH1 and RYR3 offer promising therapeutic targets, while stage-specific pathway enrichments reveal potential intervention strategies. Further validation in larger cohorts is needed to confirm these findings. Our study advances the understanding of molecular heterogeneity in ovarian cancer and lays the groundwork for personalized treatment strategies, ultimately improving patient outcomes.

## 1 INTRODUCTION

Ovarian clear cell carcinoma (OCCC) is a unique subtype of epithelial ovarian carcinoma (EOC) [1]. It is the second most common type of ovarian carcinoma after high-grade serous carcinoma (HGSC), accounting for approximately 5-10% of EOCs [2]. The advanced stage, unsatisfactory cytoreductive surgery, platinum resistance, lymph node metastasis, and venous thrombus embolism are the main factors affecting the prognosis of patients with OCCC [3]. Compared with HGSC, OCCC is significantly less sensitive to traditional chemotherapy drugs and has a high rate of resistance to platinum combined with paclitaxel chemotherapy [4, 5], a higher recurrence rate, and an unsatisfactory late prognosis [6]. Previous studies have shown a significantly better prognosis for stage I-II OCCC than for stage I-II serous carcinoma but a significantly worse prognosis for stage III-IV OCCC than for stage III-IV serous carcinoma.[7, 8] A possible underlying reason for the poorer prognosis of advanced OCCC compared with advanced serous carcinoma is the lower response rate to first -, second -, and subsequent chemotherapy [9], but further large randomized controlled trials (RCTs) are still lacking to confirm this conclusion. It is worth noting that there are few reports on the comparative analysis of the prognostic risk factors of OCCC and HGSC. Also, analysis and further study on the prognostic factors of different subgroups of OCCC and HGSC in stages I-II and III-IV are lacking [10]. At present, the treatment of advanced OCCC is still mainly based on the treatment of ovarian serous cancer [11]. Therefore, we should further explore the clinical characteristics and prognosis of stage I-II and III-IV OCCC patients compared with stage I-II and III-IV HGSC patients. It is an urgent task to obtain the clinical or molecular characteristics of OCCC and develop more effective specific treatment strategies based on its characteristics.

Whole exome sequencing (WES) has emerged as a powerful tool for identifying genetic mutations in cancer, providing insights into disease mechanisms and potential therapeutic targets [12]. Recent studies using WES have revealed distinct molecular profiles of OCCC. For instance, Yang *et al.* analyzed 42 formalin-fixed paraffin-embedded (FFPE) tissue samples of OCCC and identified frequent mutations in MUC4 (28.6%), MAGE family member E1 (MAGEE1) (19%), and AT-rich interaction domain 3A (ARID3A) (16.7%), with MAGEE1 mutations associated with poor survival outcomes [13]. Unlike HGSC, OCCC showed little association with family history, and germline mutations in breast cancer susceptibility gene 1/2 (BRCA1/2) are rare (2.1%) [14, 15]. Interestingly, OCCC patients with MYC-amplified tumors and concurrent loss of chromosome 13q12-q13 (including BRCA2) exhibited longer overall survival (OS) [16]. WES studies have also identified high-frequency somatic mutations in OCCC, such as ARID1A and PIK3CA mutations, which were also common in endometriosis, including AT-rich interaction domain 1A (ARID1A) [17] and phosphatidylinositol-4,5-bisphosphate 3-kinase catalytic subunit alpha (PIK3CA) mutations [18], which were similar to those in endometriosis [19]. Notably, PIK3CA mutation was linked to worse OS in stage I OCCC, while SWItch/sucrose non-fermentable (SWI/SNF) gene mutation correlated with better OS in advanced stages (II/III/IV) [20]. Additionally, hypoxia inducible factor-1 (HIF-1) activation and defects in the mismatch repair (MMR) system were frequently observed in OCCC [21, 22]. Despite these findings, the molecular classification of OCCC remains unclear, and the mechanisms underlying its tumorigenesis are still in the early stages of exploration.

The Surveillance, Epidemiology, and End Results (SEER) database can provide us with a large number of clinical and prognostic data on OCCC and HGSC patients. In this study, we compared the clinical characteristics, OS, and prognostic factors of OCCC and HGSC patients by extracting data from the SEER database. Our findings aim to provide a theoretical foundation for individualized clinical diagnosis, treatment strategies, and prognosis evaluation. Moreover, to further explore the reasons for the difference in prognosis between OCCC and HGSC in different stages, we utilized WES to detect tissue samples from OCCC and HGSC patients to obtain mutated genes in OCCC and HGSC, and screen for significantly mutated genes (SMGs). Tumor mutation burden (TMB), pathway enrichment analysis, and potential target drug prediction were performed for stage I-II OCCC, III-IV OCCC, I-II HGSC, and III-IV HGSC, respectively. This study was to clarify the reasons for the difference in prognosis between OCCC and HGSC of different stages.

## 2 MATERIALS AND METHODS

### 2.1 Data and Patient Source

This study compared molecular and clinical profiles between OCCC and HGSC. HGSC was selected as a reference due to its distinct molecular profile and clinical behavior, enabling subtype-specific comparisons.

Cases of primary simple OCCC and HGSC were downloaded from the SEER database (http://seer.cancer.gov/) using SEER*Stat software. We used the following criteria for inclusion and exclusion. Inclusion criteria: (1) data of cases diagnosed between January 1, 1992, and December 31, 2019, in the SEER database; Data download deadline: December 20, 2023. (2) complete tumor information, including tumor diameter, primary tumor location, *etc*. (3) histological type: patients with ICD-O-3 = "8310/3, 8313/3, 8443/3, 8444/3" or ICD-O-3 = "8441/3, 8460/3, 8461/3"and pathological diagnosis of primary simple OCCC or HGSC. (4) Detailed survival months and death outcomes were available. Exclusion criteria: (1) incomplete tumor staging data; (2) the patient's race was unknown; (3) the survival time and outcome of the patients were unknown. (4) ovarian cancer of mixed or other histological type; (5) unknown histological grade; (6) survival time less than 1 month; (7) non-primary cancer. Data in the SEER database was accessed by signing the SEER data study agreement, and the data does not contain any personally identifiable information; thus, the SEER data in this study were exempt from institutional review board examination.

The clinical samples used for WES were FFPE tissue from 15 OCCC and 16 HGSC patients admitted to the Department of Gynecology of the First Affiliated Hospital of Guangxi Medical University from October 15, 2018, to March 21, 2022. The sample size was determined by balancing biological replicate requirements and practical constraints (cost and technical feasibility). Each subgroup contained ≥ 6 biological replicates to minimize variability, and the total cohort (15 OCCC, 16 HGSC) aligns with prior exploratory studies [12, 13]. OCCC group inclusion criteria: (1) primary simple tumor confirmed by pathology; (2) initial treatment in hospital and access to pathological specimens; (3) no neoadjuvant chemotherapy and completion of standard treatment; (4) no other primary malignant tumors or serious complications affecting survival; (5) the postoperative follow-up was complete. OCCC group exclusion criteria were as follows: (1) combined with HIV, syphilis, hepatitis, and other infectious diseases; (2) patients with other types of tumors; (3) the ovarian tumor was metastatic. HGSC group inclusion/exclusion criteria: mirroring the OCCC group to ensure comparability. The study adhered to the principles of the Declaration of Helsinki, ethics approval was obtained from the hospital (2023-CR-096), and all patients provided written informed consent.

### 2.2 Data Acquisition

The variables extracted from the SEER database were as follows: (1) demographic information: patient ID, race (white, other race), age at diagnosis, the time of diagnosis; (2) clinical characteristics of the subjects: primary side (unilateral or bilateral ovarian tumors), stage (the stage was transformed from the TMN stage of the American Joint Committee on Cancer (AJCC) to the International Federation of Gynecology and Gynecology Obstetrics and Gynecology (FIGO) stage) [23], tumor size; (3) treatment information: patients were divided into 2 groups according to the chemotherapy (including neoadjuvant chemotherapy, postoperative adjuvant chemotherapy, *etc*.): receiving chemotherapy group and not receiving/unknown group; (4) clinical endpoint: OS, defined as the time from diagnosis to the date of death from any cause or the date of last follow-up, survival status (alive or dead) at the last follow-up, with data considered to be censored if the patient was still alive at the last follow-up.

The information collected from 15 cases of OCCC and 16 cases of HGSC from our hospital included age at diagnosis, tumor size, stage, tumor side, surgery, chemotherapy, and survival status.

### 2.3 WES Step

Agilent SureSelect Human All Exon V6 was selected as the exon capture kit in this study. Sequencing libraries were generated using the Truseq Nano DNA HT Sample Prep Kit (Illumina USA). Illumina NovaSeq system (Shanghai Meida Biomedical Technology Co., LTD.) was selected as the sequencing platform. The sequencing workflow was as follows: first, exon capture and genomic DNA library preparation. Cetyltrimethylammonium Bromide (CTAB) was used to extract tissue DNA. The quality of the isolated genomic DNA was verified by a combination of the following two methods: monitoring of DNA degradation and contamination on a 1% agarose gel and detection of DNA concentration with a NanoDropTM 2000/2000c spectrophotometer. Only high-quality DNA samples (OD260/280 = 1.8-2.0, OD260/230 ≥ 2.0) were used to construct sequencing libraries. Sequencing libraries were generated using the Truseq Nano DNA HT Sample Prep Kit (Illumina USA). The second step was sequencing. After the genomic DNA of the samples was detected, the DNA sequence was fragmented into random fragments by ultrasound, and the fragmented DNA was end-repaired, 3 'end added with A, and connected with a connector to prepare a DNA library. The constructed library was hybridized and captured by the Agilent SureSelect liquid phase kit. Then the target exon sequence was obtained by magnetic beads adsorption and elution, and the sequencing library was formed by PCR amplification. The constructed libraries were subjected to library quality inspection, and the qualified libraries were sequenced on the Illumina NovaSeq platform. The sequencing strategy was Illumina PE150, and the total sequencing read length was 300bp.

### 2.4 WES Data Analysis and TMB Analysis

After Illumina NovaSeq sequencing data (Raw Data) was downloaded, quality control was performed on OCCC and HGSC tumor samples to filter low-quality data to obtain high-quality data (clean data). BWA-MEME software was used to align the clean data to the reference genome sequence to obtain the position attribution of the sequence (BAM file). Subsequently, somatic gene mutation was detected based on the Mutect2 module of GATK software, and the whole exon sequencing data of normal ovarian tissue from the gnomAD database were used as a control [24-26]. These include single-nucleotide variation (SNV) and insertion-deletion (InDel) of gene sequence. The Best Practices process of GATK was used to process the alignment results (BAM file), and the Haplotyper method of GATK was used for SNV and InDel detection and filtering. The detected SNPs and InDels were functionally annotated using the VEP program [27], and the location and function of each variant locus were statistically analyzed according to the distribution of genes and functional regions of the genome.

SMGs refer to genes with a mutation frequency significantly higher than the background mutation rate. Statistical tests for individual mutation types (SMGs test) were performed with the use of Genome MuSiC and MuSiC2 software [28], with all or some tumor samples as background. The MuSic software was used to perform the convolution test, Fisher's combined *P*-value test (FCPT), and likelihood ratio test (LRT) to perform the SMGs test. Mutated genes whose presence of mutations was detectable in all collected samples were considered SMGs. Estimation of SMGs druggability was performed by accessing data from the DGIdb database (https://dgidb.org/), which includes gene-drug interaction information [29]. TMB was defined as the average number of SNVs and InDels per megabase in tumor tissue, that is, the number of somatic nonsynonymous mutations per megabase pair in the gene region detected by WES. TMB was calculated and analyzed according to stage I-II OCCC, stage III-IV OCCC, stage I-II HGSC, and stage III-IV HGSC to explore the role of TMB in tumor prognosis.

### 2.5 Enrichment Analysis of SMGs

Kyoto Encyclopedia of Genes and Genomes (KEGG) pathway enrichment analysis was performed on the SMGs of I-II OCCC, III-IV OCCC, I-II HGSC, and III-IV HGSC, respectively.

### 2.6 Statistical Analysis

Statistical analyses were performed with R software (version 4.0.5, https://www.r-project.org/) and IBM SPSS Statistics 26.0 software. The Maxstat algorithm was used to calculate the best cut-off values of continuous variables (age and tumor size) in this study [30]. Categorical data were expressed as n (%) and analyzed using the Chi-square test or Fisher's exact test. The Kaplan-Meier survival analysis method was used to draw the survival curve, and the differences between groups were tested by the log-rank test. Univariate and multivariate Cox regression analyses of the data were performed based on R software (with the packages "rms," "foreign," and "survival"), and *P* values of less than 0.05 were considered to be statistically significant in all analyses.

## 3 RESULTS

### 3.1 Key Differences in Clinical Characteristics Between OCCC and HGSC Patients in the SEER Database

The number of patients with OCCC and HGSC selected from the SEER database was 3493 and 10266, respectively. Table **1** summarizes the clinical characteristics of patients in the SEER database. The cut-off values of continuous variables such as age and tumor size calculated by the Maxstat algorithm were 60 years and 87mm, respectively. Therefore, the age group was divided into < 60 years and ≥ 60 years groups. Tumor size was divided into < 87mm and ≥ 87mm groups.

White people accounted for 72.6% (2682/3693) in the total OCCC group, 27.4% (1011/3693) in the other race groups, and 84.6% (868/10266) in the HGSC group. The proportion of patients aged ≥ 60 years in the overall OCCC group was significantly lower than that in the overall HGSC group. The proportion of unilateral tumor distribution in the OCCC and HGSC groups was 85.5% (3158/3693) and 39.8% (4087/10266), respectively (*P* < 0.05). There was a significant difference in disease stage between the two groups. 69.1% (2552/3693) of the OCCC patients were stage I-II, while 15.8% (1627/10266) of the HGSC patients were stage I-II. The proportion of patients with tumor size ≥ 87mm in the OCCC group was significantly higher than that in the HGSC group, *P* < 0.05, as shown in Table **2**.

The proportion of patients aged ≥ 60 years in the I-II stage OCCC group was lower than that in the I-II stage HGSC group (*P* < 0.05). The proportion of unilateral tumor distribution was 95.1% (2426/2552) and 66.9% (1089/1627), respectively. The proportion of patients with tumor size ≥ 87mm in the OCCC group was significantly higher than that in the HGSC group in stage I-II, *P* < 0.05, as shown in Table **2**. These trends were also observed for the baseline characteristics of the stage III-IV OCCC group versus the stage III-IV HGSC group (Table **3**).

### 3.2 OCCC Has Better Early-Stage but Worse Advanced-Stage Survival vs. HGSC

The 3-year and 5-year survival rates of overall OCCC patients were 70.0% and 63.5%, while the 3-year and 5-year survival rates of overall HGSC patients were 55.5% and 37.0%, which illustrated that the prognosis of OCCC was significantly better than that of HGSC, *P* < 0.05 (Fig. **1A**). The 3-year and 5-year survival rates of stage I-II OCCC patients were 87.2% and 79.5%, while the 3-year and 5-year survival rates of patients with stage I-II HGSC were 83.0% and 70.8%, which indicated OS of stage I-II OCCC was significantly better than that of stage I-II HGSC, *P* < 0.05 (Fig. **1B**). The 3-year and 5-year survival rates of stage III-IV OCCC patients were 35.5% and 25.3%, the 3-year and 5-year survival rates of stage III-IV HGSC patients were 50.2% and 30.0%, which showed that the OS of stage III-IV OCCC was significantly worse than that of stage III-IV HGSC, *P* < 0.05 (Fig. **1C**).

### 3.3 Prognostic Factors in OCCC and HGSC: Pathological Type, Age, Tumor Features, and Treatment Influence Survival

After grouping according to survival status, univariate analysis of patient clinical variables in stage I-II OCCC and HGSC patients showed that pathological type, age, race, laterality, tumor size, stage, and whether to receive chemotherapy were associated with OS of stage I-II patients, *P* < 0.05 (Table **4**). For stage I-II patients, those with a pathological type of HGSC, age ≥ 60 years, bilateral tumor involvement, tumor diameter ≥ 87mm, stage II, and no chemotherapy had a worse prognosis, and those with other races had a better prognosis. For stage III-IV OCCC and HGSC patients, patients with age ≥ 60 years, bilateral tumor involvement, stage IV, and without chemotherapy had a worse prognosis, and patients with the pathological type of HGSC and tumor size ≥ 87mm had a better prognosis (Table **5**).

### 3.4 Stage-Specific Prognostic Factors in Overall OCCC and HGSC Revealed by Multivariate Analysis

Variables with statistically significant differences in univariate analysis were included in multivariate COX regression analysis. The results showed that pathological type of HGSC, age ≥ 60 years, bilateral tumor involvement, tumor size ≥ 87mm, stage II, and no chemotherapy were independent risk factors for the prognosis of patients with overall stage I-II OCCC and HGSC (*P* < 0.05) (Fig. **2A**). The results showed that age ≥ 60 years, bilateral tumor involvement, stage IV, and no chemotherapy were independent risk factors for the prognosis of patients with overall stage III-IV OCCC and HGSC. It is worth noting that the pathological type of HGSC and tumor size ≥ 87mm were independent protective factors for the prognosis of overall stage III-IV OCCC and HGSC (*P* < 0.05) (Fig. **2B**).

### 3.5 Independent Risk and Protective Factors for Survival in OCCC and HGSC

Univariate analysis showed that for patients with OCCC, age, laterality, tumor size, and stage were significantly associated with the patient's OS (*P* < 0.05) (Table **6**). For HGSC patients, age, side, tumor size, stage, and whether to receive chemotherapy were significantly associated with the OS of HGSC patients (Table **7**).

Multivariate analysis showed that age ≥ 60 years, bilateral tumor involvement, stage III-IV, and tumor size ≥ 87 mm were independent risk factors for prognosis in patients with OCCC (Fig. **3A**). For HGSC patients, age ≥ 60 years, bilateral tumor involvement, stage III-IV, and no chemotherapy were independent risk factors for prognosis (*P* < 0.05). However, tumor size ≥ 87mm was an independent protective factor for the prognosis of HGSC patients (Fig. **3B**).

### 3.6 Clinical Characteristics and Follow-Up Outcomes of Hospital-Collected OCCC and HGSC Patients

One stage IV OCCC sample was excluded from the FFPE samples obtained from our hospital after strict quality control, and the remaining samples were of high quality. The clinical data of 15 cases of OCCC and 16 cases of HGSC are shown in Table **8**. The mean age at first diagnosis was 49.5 years (range from 36 to 61 years) for OCCC and 53.1 years (range from 36 to 70 years) for HGSC, with no significant difference in tumor size between the groups. All patients received radical surgery, and one patient did not receive chemotherapy due to the early stage. By February 2024, the follow-up time ranged from 18 to 115 months. Three patients in OCCC died after 61, 78, and 45 months of follow-up, respectively, all of whom were stage III-IV, and two patients in HGSC died after 68 and 71 months of follow-up, both of whom were stage I-II, and the rest were still alive.

### 3.7 Somatic Mutations and TMB in OCCC and HGSC: Stage-Specific Patterns and Trends

Mutational characteristics of the sequencing data of 8 cases of stage I-II OCCC (A1-A8), 7 cases of stage III-IV OCCC (A9-A15), 8 cases of stage I-II HGSC (B1-B8), and 8 cases of stage III-IV HGSC (B9-B16) were summarized in Table **9**. Due to the long storage time of the paraffin blocks collected in our hospital and the technical problems of intraoperative frozen pathological processing, the sampling and quality inspection of normal adjacent tissues were not satisfactory. The raw sequencing data were processed and compared with the WES data of normal ovarian tissues in the gnomAD database to obtain the somatic gene mutation results [25], as shown in Table **9**. The main mutation type of somatic SNV in OCCC and HGSC is a missense mutation, and the most common mutation in somatic InDel of OCCC and HGSC is a frameshift mutation. The frequency of SNVs varied greatly among different samples. The reason for this difference may be that some samples were stored for a long time, leading to damage and degradation of nucleic acids. Most of the transitions between bases were C→T (cytosine mutated to thymine) transitions, followed by T→C (thymine mutated to cytosine) transitions (Fig. **4A**-**D**).

The distribution of TMB in 15 OCCC and 16 HGSC patients is shown in Table **9**. The mean TMB was 408.74 Mut/Mb in 8 cases of stage I-II OCCC, 268.45 Mut/Mb in 7 cases of stage III-IV OCCC, 395.63 Mut/Mb in 8 cases of stage I-II HGSC, and 308.83 Mut/Mb in 8 cases of stage III-IV HGSC. The TMB of stage I-II OCCC was numerically higher than that of stage I-II HGSC, and the TMB of stage III-IV OCCC was numerically lower than that of stage III-IV HGSC, but neither difference was statistically significant (*P* > 0.05) (Fig. **5**).

 A1-A8: 8 cases of stage I-II OCCC; A9-A15: 7 cases of stage III-IV OCCC; B1-B8: 8 cases of stage I-II HGSC; B9-B16: 8 cases of stage III-IV HGSC. Missense Variant: number of SNV sites of the missense mutation in the exon region of the sample; Start Lost: Number of SNV sites of the promoter deletion due to the SNP mutation; Stop Gained: Number of SNV sites of the terminator due to the SNV mutation; Stop Lost: Synonymous Variant: The number of SNV sites with synonymous mutations in the exon region of the sample; Frameshift Variant: The number of InDels causing the Frameshift mutation.

### 3.8 Stage-Specific and Shared SMGs in OCCC and HGSC Reveal Distinct Molecular Profiles

A total of 272 SMGs were identified in stage I-II OCCC, 324 SMGs were identified in stage I-II HGSC, and 58 SMGs were common between the two groups (Fig. **6A**). In stage III-IV, OCCC showed 244 SMGs versus 139 in HGSC, sharing 42 SMGs (Fig. **6B**). The total number of mutations in the top 20 SMGs ranged from 68 to 365 in stage I-II 8 OCCC patients, and the most common mutation type was missense mutation, followed by nonsense mutation (Fig. **7A**). Among the top 20 SMGs, 10 genes including BSN, HERC2, HMCN2, LRP1, MUC19, PLEC, RNF213, RYR1, RYR3, and PTBN5 were identified as stage I-II OCCC-specific SMGs. In stage I-II HGSC, the total number of mutations in the top 20 SMGs was high (83-453) (Fig. **7B**). ADGRV1, CCDC168, FAT4, HERC1, KMT2D, MACF1, NEB, STARD9, SYNE2, ZNF469, and 10 other genes are unique SMGs that distinguish stage I-II HGSC from stage I-II OCCC. Among the top 20 SMGs cohorts, the following 10 mutated genes were considered common SMGs in stage I-II OCCC and stage I-II HGSC, such as AHNAK2, DNAH1, DST, MUC16, MUC5B, OBSCN, PKD1, SPEG, SYNE1, and TTN (Fig. **7B**).

Waterfall plot (Fig. **7C** and **D**) illustrated the mutational landscape of the top 20 SMGs in the stage III-IV OCCC and HGSC, alongside subtype-specific variations from 7 stage III-IV OCCC and 8 stage III-IV HGSC cases. The main mutation type was a missense mutation, followed by a nonsense mutation. The total number of mutations in the top 20 SMGs fluctuated between 52 and 280 in stage III-IV OCCC (Fig. **7C**). Among the top 20 SMGs in stage III-IV OCCC, DNAH2, LAMA5, MUC19, NOTCH1, PCLO, SYNE2, TACC2, and ZNF469 were eight specific SMGs that distinguished stage III-IV HGSC. In 8 cases of stage III-IV HGSC, the total number of mutations in the top 20 genes was high (57-334). Among them, eight SMGs, including DNAH17, DYNC1H1, HERC2, HMCN2, MUC5AC, RYR3, SPEG, and SYNE1, were distinguished between stage III-IV HGSC and stage III-IV OCCC (Fig. **7D**). Among the top 20 SMGs, the following 12 mutated genes were the same in stage III-IV OCCC and stage III-IV HGSC cohorts, including ADGRV1, AHNAK, AHNAK2, HSPG2, MACF1, MUC16, MUC5B, NEB, OBSCN, PLEC, RYR1, and TTN.

The top bar shows the sample’s TMB, while the right bar indicates mutation frequency per gene. Different colors denote mutation types, with columns representing patients and rows representing genes.

### 3.9 Pathway Enrichment Highlights Stage-Specific Molecular Differences in OCCC and HGSC

KEGG analysis was performed on 214 stage I-II OCCC-specific SMGs and 266 stage I-II HGSC-specific SMGs. The results suggested that the common main enriched pathway was the cyclic guanosine monophosphate-protein kinase(cGMP-PKG) signaling pathway (Fig. **8A** and **B**). SMGs in stage I-II OCCC were mainly enriched in calcium signaling pathway and extracellular matrix (ECM) receptor interaction pathway (Fig. **8A**). However, SMGs in stage I-II HGSC were mainly enriched in signaling pathways such as ABC transporters, tight junctions, and base excision repair (Fig. **8B**). The 202 stage III-IV OCCC-specific SMGs and 97 stage III-IV HGSC-specific SMGs were enriched in the following two pathways: Human papillomavirus infection and ECM receptor interaction pathway (Fig. **8C** and **D**). SMGs in stage III-IV OCCC were mainly enriched in the Notch signaling pathway, focal adhesion, and other pathways (Fig. **8C**). However, the SMGs of stage III-IV HGSC were mainly enriched in lysine degradation, protein digestion and absorption, apelin signaling pathway, phospholipase D signaling pathway, and longevity regulation pathway (Fig. **8D**).

### 3.10 Targetable Genes and Therapeutic Opportunities in Stage III-IV OCCC and HGSC

Among the Top 20 SMGs found in stage III-IV OCCC and stage III-IV HGSC samples, 9 SMGs have been described in the DGIdb database as targetable by known drugs. NOTCH1 in stage III-IV OCCC can be targeted by 7 drugs. SYNE2 was targeted by one drug; LAMA5 was targeted by one drug. In stage III-IV HGSC, RYR3 could be targeted by 8 drugs, MUC5AC by 4 drugs, and HERC2 by one drug. In stage III-IV OCCC and stage III-IV HGSC cohorts, three common mutated genes, including MUC16, could be targeted by 14 drugs, RYR1 could be targeted by 14 drugs, and 3 drugs could target HSPG2. Drug-gene interaction information is detailed in Table **10**. Among the drugs that have already obtained regulatory approval, several are targeted therapies, including neratinib and tamoxifen. Neratinib targets the HER2/EGFR pathway [31], while tamoxifen acts on the estrogen receptor [32]. Both are used to treat specific molecular subtypes of gynecological tumors. In the realm of immunotherapy, oregovomab and abagovomab target CA125 and are primarily used for treating ovarian cancer [23, 33]. For chemotherapy, vincristine, topotecan, docetaxel, and etoposide are the mainstays [34]. These drugs work by inhibiting microtubules or topoisomerases and are widely used in the treatment of ovarian cancers. On the other hand, cyclosporine and palifermin serve as immune regulators, which are primarily used for supportive therapy to mitigate the side effects of chemotherapy or radiotherapy [35]. By targeting specific pathways or molecules, these predicted drugs have played important roles in treating OCCC and HGSC.

## 4 DISCUSSION

Based on SEER data, OCCC patients showed significantly better OS than HGSC patients, particularly in stage I-II; however, stage III-IV OCCC had worse OS compared to HGSC, aligning with prior Chinese studies [36]. Compared with patients with serous cancer, the OS of patients with advanced OCCC was significantly worse (38.0 *vs* 52.0 months; HR = 1.584; 95%CI: 1.17-2.15, *P* = 0.003) [36]. In addition, data from 12 prospective randomized GOG trials suggested that the prognosis of patients with advanced OCCC was significantly worse than that of patients with serous carcinoma, with progression-free survival (PFS) and OS of advanced OCCC being lower than that of advanced serous carcinoma (HR = 1.66, 95%CI: 1.43-1.91) [9]. A study conducted in Korea on trends in incidence and survival comparing ovarian serous cancer and OCCC observed that OCCC patients with distant disease had worse OS than serous patients [37]. Similarly, according to a previous meta-analysis, patients with stage III-IV OCCC (n = 845) had a higher risk of death than patients with stage III-IV serous adenocarcinoma (n = 18,471; HR = 1.71, 95%CI: 1.57-1.86) [38].

This study identified three common independent risk factors for OCCC and HGSC: age ≥ 60 years, bilateral tumors, and III-IV stage. Tumor size ≥ 87mm was a risk factor in OCCC but a protective factor in HGSC, aligning with previous findings [36]. However, it is still a controversial issue to use tumor size to assist tumor staging and predict prognosis and treatment response in EOC. Recently, Wu *et al.* established the OCCC database for retrospective analysis by extracting original clinical and pathological information from 1998 to 2018 through the SEER database [39]. In 1000 patients with stage I OCCC, the prognosis of patients with tumor diameter ≤ 8cm was significantly better than that of patients with tumor diameter > 8cm (HR = 0.5608, 95%CI: 0.41-0.76, *P* = 0.0002). The 5-year OS rate of patients with tumor size > 8cm was 80.6%, while that of patients with tumor size ≤ 8cm was 90.2% (*P* < 0.0001) [39]. For serous tumors, the situation is more complex. Previously, Paik *et al.* found that advanced serous carcinomas with smaller tumors had a worse prognosis than those with larger tumors [40]. Meanwhile, Horvath *et al*. [41] found larger tumor volumes in early vs. advanced EOC, while another study of 553 patients showed larger volumes in stage III vs. stage I EOC, linking poor prognosis to abdominal metastasis size [42]. Despite controversial tumor size roles in EOC prognosis, multivariate COX analysis in our study revealed tumor size ≥ 87mm as an independent protective factor for HGSC, highlighting its prognostic value.

To explore the prognostic differences between the two groups, we used WES and identified specific SMGs distinguishing stage III-IV OCCC from stage III-IV HGSC: zinc finger protein 469 (ZNF469), spectrin repeat containing nuclear envelope protein 2 (SYNE2), piccolo presynaptic cytomatrix protein (PCLO), transforming acidic coiled-coil containing protein 2 (TACC2), dynein axonemal heavy chain 2 (DNAH2), laminin subunit alpha 5 (LAMA5), mucin 19 (MUC19), and neurogenic locus Notch homolog protein 1 (NOTCH1). Previous studies have shown that ZNF469 is crucial for prognosis evaluation and immune cell infiltration in esophageal cancer. Its expression level is positively correlated with CD4+ T cell infiltration and negatively correlated with patients' DFS [43]. Also, high ZNF469 expression in lung adenocarcinoma correlates with poor prognosis and impacts immune infiltration [44], but there was no related study of ZNF469 in OCCC. The SYNE2 gene encodes a cytoskeletal linker protein [45]. An in vitro study found that Nesprin-2 was localized in the nuclear envelope and nucleus of EOC cell line SKOV-3 and the cytoplasm of EOC cell line Caov-3 [46]. It plays key roles in DNA damage repair, chromatin organization, and cell migration [47]. TACC2, a centrosome spindle-binding protein, plays roles in cell division and DNA repair by interacting with the BRCA1-associated RING domain 1 (BARD1)/BRCA1 complex [48, 49]. It also binds to histone deacetylase (HDAC) and SWI/SNF complexes, regulating chromatin remodeling and transcription [50]. Single-cell RNA sequencing revealed TACC2 overexpression in fallopian tube epithelial cells, linking it to EOC risk and ovarian stromal cell overgrowth [51]. Previous studies have revealed the clinical importance of cell adhesion molecules for the diagnosis and prognosis of patients with OCCC [52]. LAMA5, a member of the laminin family, was overexpressed in endometrioid ovarian cancer [53] and OCCC [54]. The knockdown of LAMA5 could inhibit the proliferation of ovarian cancer cell lines *in vivo* and in vitro. It may play a role in the epithelial-mesenchymal transition process by acting on the Notch signaling pathway to promote the growth of ovarian cancer cells [55]. NOTCH1 mutations in head and neck squamous cell carcinoma activate the EGFR-PI3K-AKT pathway, making tumors more sensitive to PI3K/mTOR inhibitors [56, 57]. This suggests PI3K inhibitors may benefit ovarian cancer patients with lymph node metastasis, as NOTCH1 mutations may drive this process [58]. However, Crobach *et al.* found NOTCH1 mutations only in primary ovarian tumors, not in metastatic tumors [59], highlighting the need for further research on its role in OCCC.

Based on KEGG enrichment analysis, it was found that the SMGs of I-II stage OCCC and I-II stage HGSC were mainly enriched in the cGMP-PKG signaling pathway, and the basal activity of this pathway led to continuous inhibition of p53 and protection from spontaneous apoptosis [60]. An *in vitro* study showed that the basal activity of NO/cGMP/PKG signaling protects human ovarian cancer cells from spontaneous apoptosis and provides resistance to cisplatin-induced apoptosis [61]. In contrast, stage I-II HGSC SMGs were enriched in the ABC transporter pathway, linked to chemoresistance by reducing intracellular drug accumulation [62, 63], likely contributing to HGSC's worse prognosis compared to OCCC. This also indicated that I-II stage HGSC has stronger drug resistance than OCCC, which affected the prognosis of I-II stage HGSC was worse than I-II stage OCCC. Additionally, stage III-IV OCCC SMGs were enriched in the Notch pathway, associated with chemotherapy resistance and recurrence [64, 65]. A candidate gene analysis of 2 endometrioid and 8 endometriosis-associated OCCC cases suggested the Notch pathway promotes malignant transformation [66], indicating that NOTCH2 mutations may be an early event in advanced OCCC. However, the role of Notch signaling in endometriosis-to-OCCC transformation requires further study.

Previous studies suggest that higher TMB correlates with better prognosis and earlier clinical stage [67]. In this study, TMB comparisons between I-II and III-IV OCCC and HGSC showed no significant differences, but stage III-IV OCCC had numerically lower TMB than HGSC, aligning with its worse prognosis. Immunotherapy efficacy is theoretically linked to TMB, as higher TMB may generate more neoantigens, enhancing T cell recognition [68]. For instance, the CheckMate-032 trial in advanced lung cancer found high-TMB patients had significantly longer median OS (22.0 months) than medium- (3.6 months) and low-TMB groups (3.4 months) [69]. Similarly, in ovarian cancer, high TMB correlates with better prognosis and is linked to FIGO stage, grade, and residual tumor size [70].

Furthermore, by performing drug-gene interaction analysis *via* the DGIdb database, these findings can provide the rationale for drug investigation [71]. Drugs that have not received regulatory approval work through different mechanisms, such as Ro4929097 [72], Crenigacestat [73], and Mk-0752 [74], which can work as γ-secretase inhibitors to inhibit the Notch signaling pathway. In gynecologic tumors, preliminary results of these agents suggest good tolerability and potential efficacy, especially in combination therapy and precision treatment targeting specific molecular markers [75, 76]. However, the research on these drugs is still in the early stage, and more clinical trials are needed in the future to verify their efficacy and safety in gynecological tumors and to provide patients with more effective treatment options.

## 5 STUDY LIMITATIONS

The study has several limitations: 1. Reliance on retrospective SEER data may introduce inconsistencies in clinical documentation. Specifically, chemotherapy variables are limited to "yes" and "no" categories, preventing analysis of specific regimens' effects on prognosis. In contrast to prospective studies, cases drawn from the SEER database were not revised by a single pathologist and may be prone to misclassification. Additionally, SEER data primarily represent white populations, limiting insights into Asian populations. 2. The sample size, particularly for early-stage HGSC, is small due to the rarity of these cases, which may limit generalizability. The prognostic analysis of 15 OCCC and 16 HGSC patients contained extensive censored data, preventing the establishment of a relationship between TMB and prognosis. 3. The use of FFPE samples, while clinically relevant, may introduce biases due to potential DNA degradation over time. 4. The absence of functional validation for identified SMGs and pathways limits mechanistic insights from our data. 5. The study did not explore other potential mechanisms, such as epigenetics, proteomics, microbiology, and metabolism, which may contribute to the prognostic differences between OCCC and HGSC. Future studies should include prospective designs, larger cohorts, functional experiments, and multi-omics approaches to address these limitations.

Our study also has certain strengths. We excluded low-grade serous carcinoma (LGSC) and only included HGSC, which was less heterogeneous and more targeted. In addition, few studies have compared the clinical characteristics and prognostic factors of stage I-II OCCC and stage I-II HGSC, and stage III-IV OCCC and stage III-IV HGSC separately. The most important was that we used WES for the first time to detect SMGs and compared the differences of SMGs between stage I-II OCCC and stage I-II HGSC, stage III-IV OCCC, and stage III-IV HGSC, and performed enrichment analysis to find possible pathway differences.

## CONCLUSION

This study highlights significant differences in clinical characteristics, survival outcomes, and molecular profiles between OCCC and HGSC. OCCC patients showed better OS than HGSC in stage I-II, but worse OS in stage III-IV. The distinct SMGs and enriched pathways between OCCC and HGSC suggest different underlying biological mechanisms, which may serve as potential targets for molecular therapy. Notably, stage III-IV OCCC exhibited unique SMGs and pathway enrichments, offering opportunities for targeted therapies. Additionally, drug-target interaction analysis identified actionable targets for advanced OCCC and HGSC. However, further *in vivo* and *in vitro* studies are needed to validate these findings and explore their therapeutic potential.

## AUTHORS’ CONTRIBUTIONS

The authors confirm their contribution to the paper as follows: study conception and design: JF; data collection: HH; Data Analysis or Interpretation: YW; draft manuscript: XL, YY, and SZ. All authors reviewed the results and approved the final version of the manuscript.

## Figures and Tables

**Fig. (1):**
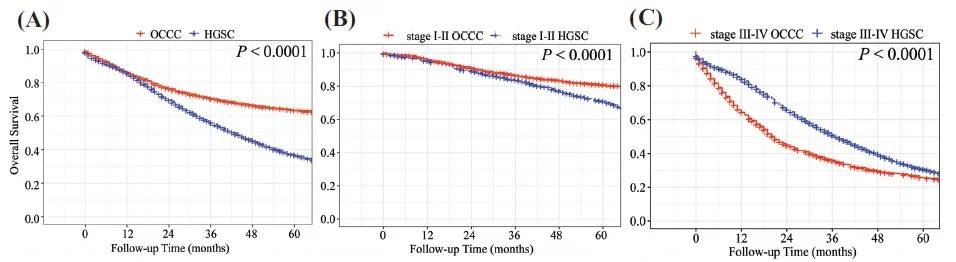
OS differences between OCCC and HGSC patients in the SEER database. OS comparison between (**A**) overall OCCC and HGSC patients, (**B**) stage I-II OCCC and HGSC patients, and (**C**) stage III-IV OCCC and HGSC patients. Red represents OCCC, and blue represents HGSC.

**Fig. (2):**
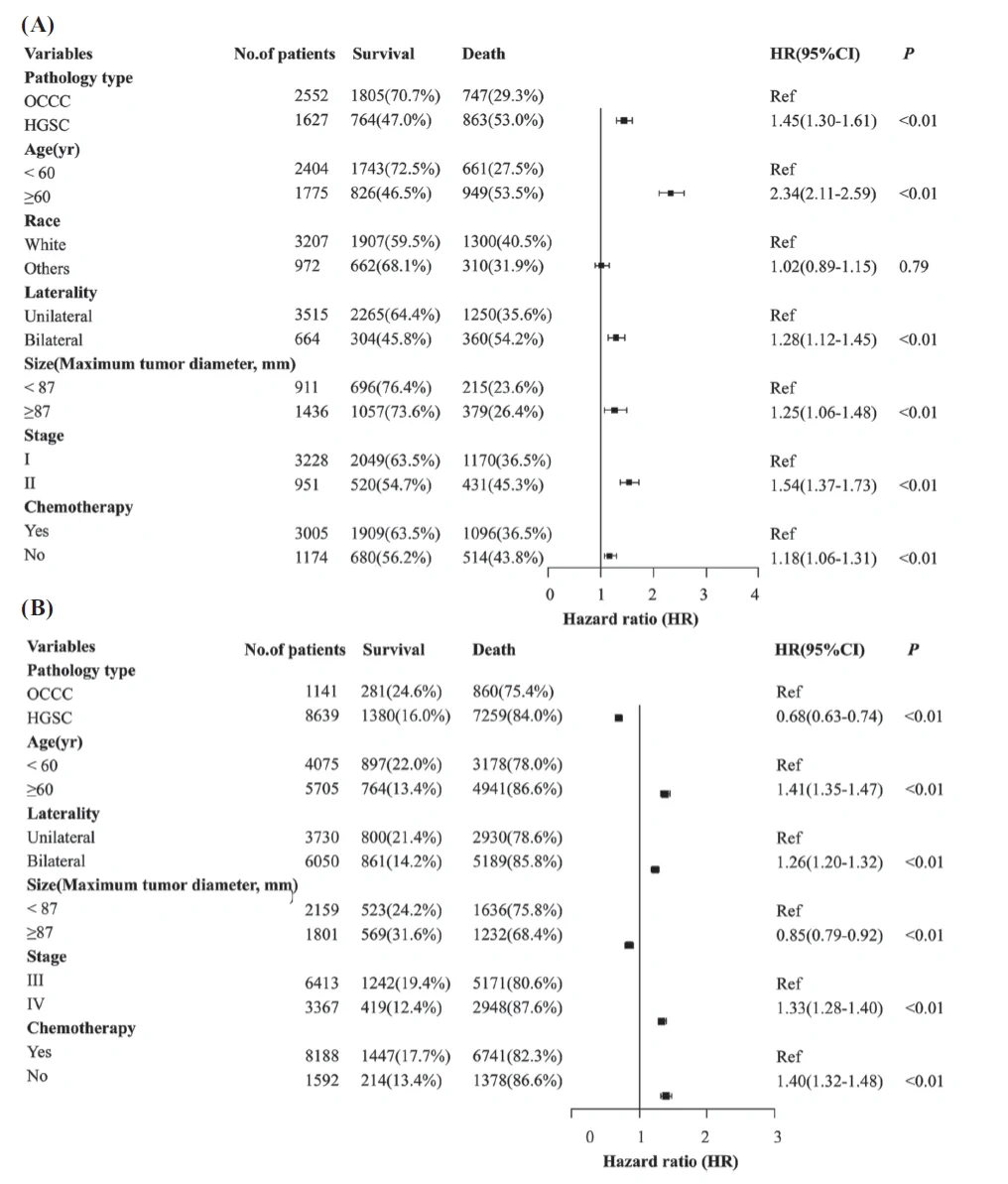
Multivariate COX regression analysis of prognostic factors in (**A**) stage I-II and (**B**)III-IV patients in the SEER database.

**Fig. (3):**
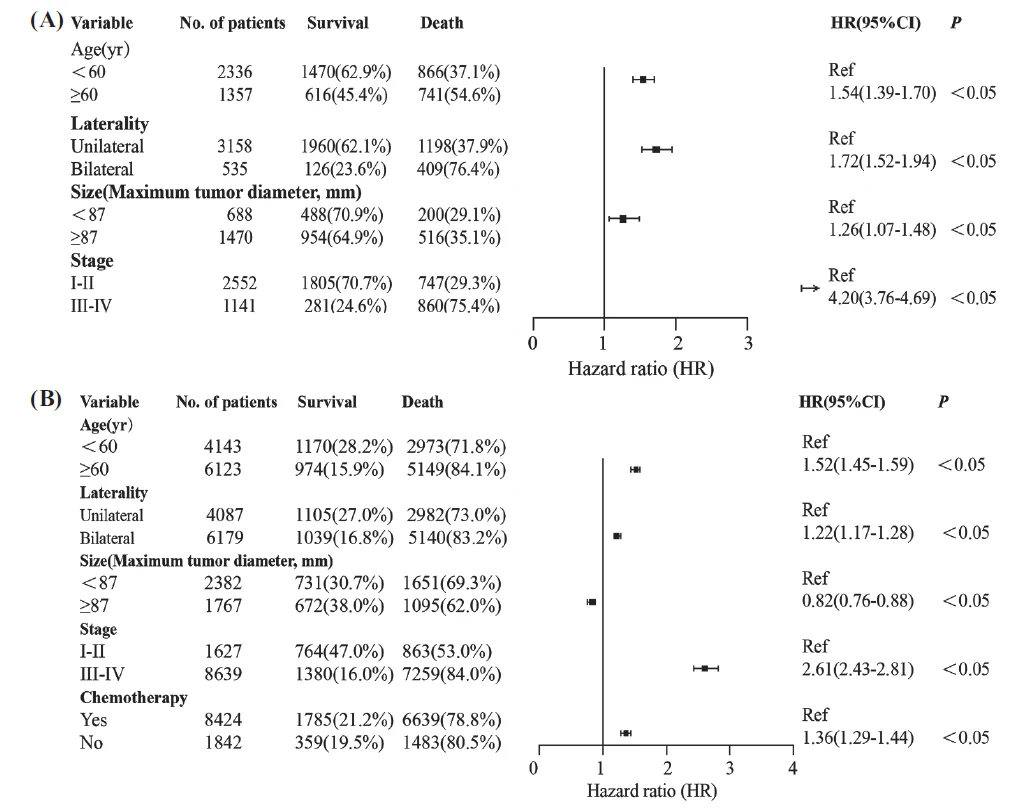
Multivariate COX regression analysis of prognostic factors for (**A**) OCCC and (**B**) HGSC patients in the SEER database.

**Fig. (4):**
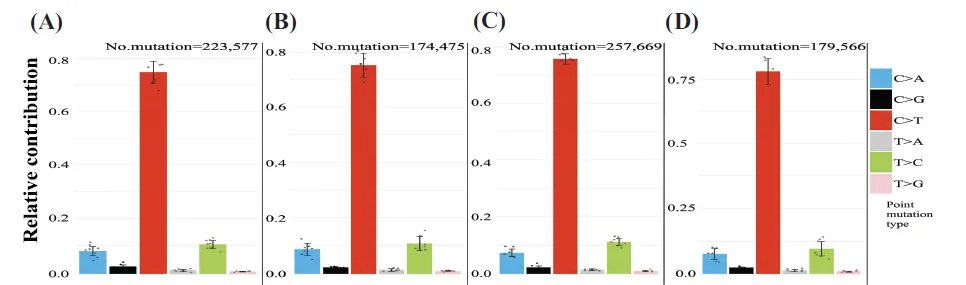
Spectrum of somatic mutations of (**A**) stage I-II OCCC, (**B**) stage I-II HGSC, (**C**) stage III-IV OCCC, and (**D**) stage III-IV HGSC.

**Fig. (5):**
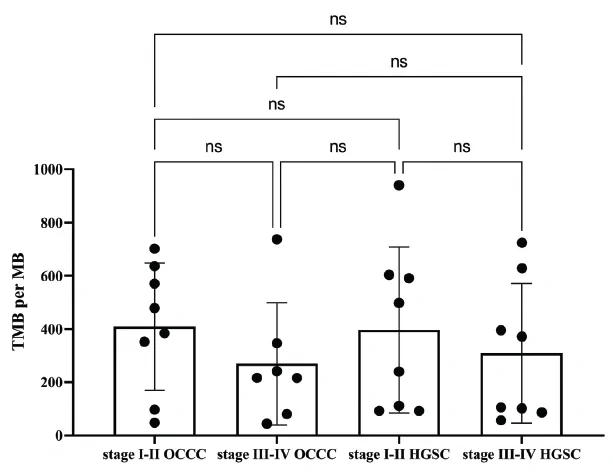
Comparison of TMB in 8 cases of stage I-II OCCC, 7 cases of stage III-IV OCCC, 8 cases of stage I-II HGSC, and 8 cases of stage III-IV HGSC group. (*A higher resolution / colour version of this figure is available in the electronic copy of the article*).

**Fig. (6):**
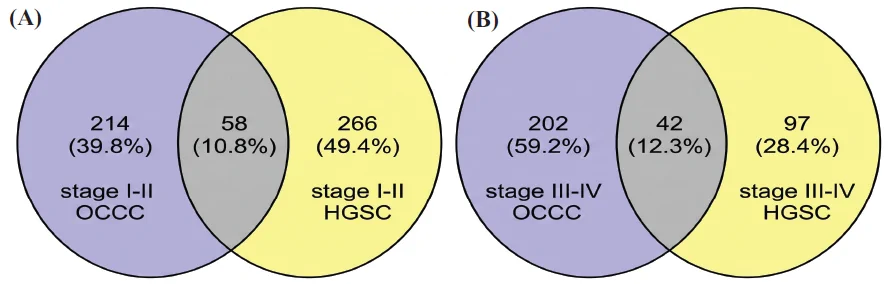
Venn diagram of frequently mutated genes in (**A**) stage I-II OCCC and HGSC, (**B**) stage III-IV OCCC and HGSC.

**Fig. (7):**
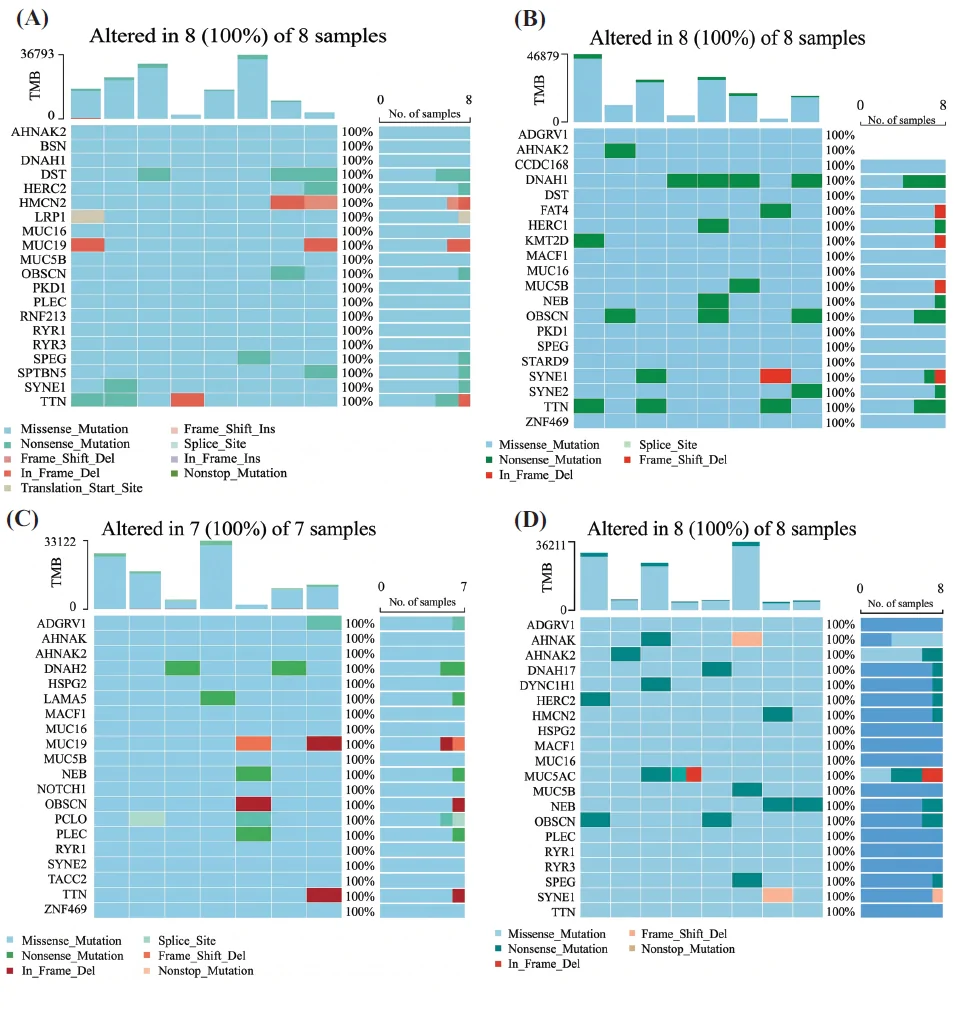
SMGs with top 20 total mutation frequency for (**A**) stage I-II OCCC, (**B**) stage I-II HGSC, (**C**) stage III-IV OCCC, and (**D**) stage III-IV HGSC.

**Fig. (8):**
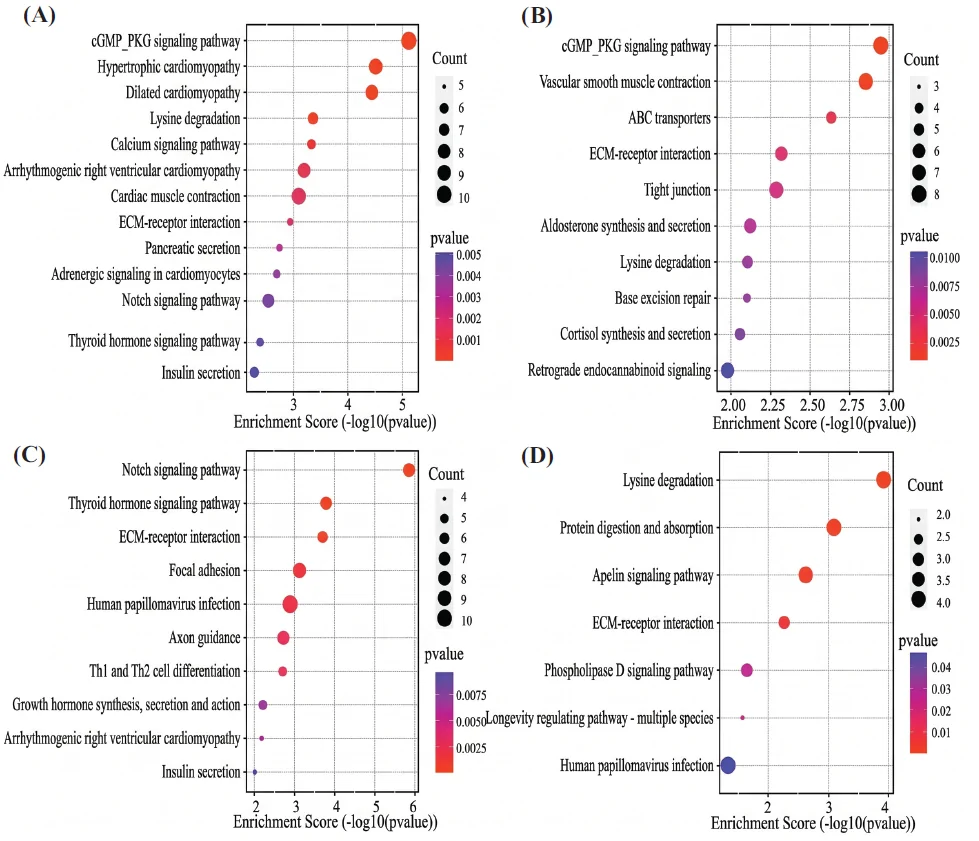
KEGG enrichment analysis of frequently mutated genes in (**A**) stage I-II OCCC, (**B**) stage I-II HGSC, (**C**) stage III-IV OCCC, and (**D**) stage III-IV HGSC.

**Table 1:** Clinical differences between OCCC and HGSC patients in the SEER database.

**Variables**		**n (%)**	**OCCC (n = 3693)**	**HGSC (n = 10266)**	***P-*Value**
Age(yr)	<60	6479 (46.4)	2336 (63.3)	4143 (40.4)	<0.05
-	≥60	7480 (53.6)	1357 (36.7)	6123 (59.6)	-
Race	White	11362 (81.4)	2682 (72.6)	8680 (84.6)	<0.05
-	Others	2594 (18.6)	1011 (27.4)	1586 (15.4)	-
Laterality	Unilateral	7245 (51.9)	3158 (85.5)	4087 (39.8)	<0.01
-	Bilateral	6714 (48.1)	535 (15.5)	6179 (60.2)	-
Size(mm)	<87	3070 (22.0)	688 (18.6)	2382 (23.2)	<0.05
-	≥87	3237 (23.2)	1470 (39.8)	1767 (17.2)	-
-	NA	7652 (54.8)	1535 (41.6)	6117 (59.6)	-
Stage	I-II	4179 (29.9)	2552 (69.1)	1627 (15.8)	<0.05
	III-IV	9780 (70.1)	1141 (30.9)	8639 (84.2)	-
Chemotherapy	Yes	11193 (80.2)	2769 (75.0)	8474 (82.5)	<0.05
-	No	2766 (19.8)	924 (25.0)	1842 (17.9)	-

**Table 2:** Clinical differences between the stage I-II OCCC and HGSC groups in the SEER database.

**Variables**		**n (%)**	**Stage I-II OCCC** **(n = 2552)**	**Stage I-II HGSC** **(n = 1627)**	***P-*Value**
Age(yr)	<60	2404 (57.5)	1681 (65.9)	723 (44.4)	<0.05
-	≥60	1775 (42.5)	871 (34.1)	904 (55.6)	-
Race	White	3207 (76.7)	1844 (72.3)	1363 (83.8)	<0.05
-	Others	972 (23.3)	708 (27.7)	264 (16.2)	-
Laterality	Unilateral	3515 (84.1)	2426 (95.1)	1089 (66.9)	<0.05
-	Bilateral	664 (15.9)	126 (4.9)	538 (33.1)	-
Size(mm)	<87	911 (21.8)	518 (20.3)	393 (24.2)	<0.05
-	≥87	1436 (34.4)	1037 (40.6)	399 (24.5)	-
-	NA	1832 (43.8)	997 (39.1)	835 (51.3)	-
Stage	I	3228 (77.2)	2153 (84.4)	1075 (66.1)	<0.05
	II	951 (22.8)	399 (15.6)	552 (33.9)	-
Chemotherapy	Yes	3005 (71.9)	1830 (71.7)	1175 (72.2)	0.07
-	No	1174 (28.1)	722 (28.3)	452 (27.8)	-

**Table 3:** Clinical differences between the stage III-IV OCCC and HGSC groups in the SEER database.

**Variables**		**n (%)**	**Stage III-IV OCCC** **(n = 1141)**	**Stage III-IV HGSC** **(n = 8639)**	***P-*Value**
Age(yr)	<60	4075 (41.7)	655 (57.4)	3420 (39.6)	<0.05
-	≥60	5705 (58.3)	486 (42.6)	5219 (60.4)	-
Race	White	8155 (83.4)	838 (73.4)	7317 (84.7)	<0.05
-	Others	1625 (16.6)	303 (26.6)	1322 (15.3)	-
Laterality	Unilateral	3730 (38.1)	732 (64.2)	2998 (34.7)	<0.05
-	Bilateral	6050 (61.9)	409 (35.8)	5641 (65.3)	-
Size (mm)	<87	2159 (22.1)	170 (14.9)	1989 (23.0)	＜0.05
-	≥87	1801 (18.4)	433 (37.9)	1368 (15.8)	-
-	NA	5820 (59.5)	538 (47.2)	5282 (61.2)	-
Stage	III	6413 (65.6)	772 (67.7)	5641 (65.3)	0.11
-	IV	3367 (34.4)	369 (32.3)	2998 (34.7)	-
Chemotherapy	Yes	8188 (83.7)	939 (82.3)	7249 (83.9)	0.17
-	No	1592 (16.3)	202 (17.7)	1390 (16.1)	-

**Table 4:** Univariate analysis of stage I-II OCCC and HGSC prognosis.

**Variables**		**No. of Patients**	**Survival (n)**	**Death (n)**	**HR (95%CI)**	***P-*Value**
Pathology type	OCCC	2552	1805 (70.7%)	747 (29.3%)	Ref	-
-	HGSC	1627	764 (47.0%)	863 (53.0%)	1.89 (1.72-2.09)	<0.05
Age (yr)	<60	2404	1743 (72.5%)	661 (27.5%)	Ref	-
-	≥60	1775	826 (46.5%)	949 (53.5%)	2.58 (2.34-2.86)	<0.05
Race	White	3207	1907 (59.5%)	1300 (40.5%)	Ref	-
-	Others	972	662 (68.1%)	310 (31.9%)	0.83 (0.73-0.94)	<0.05
Laterality	Unilateral	3515	2265 (64.4%)	1250 (35.6%)	Ref	-
-	Bilateral	664	304 (45.8%)	360 (54.2%)	1.67 (1.49-1.88)	<0.05
Size(mm)	<87	911	696 (76.4%)	215 (23.6%)	Ref	-
-	≥87	1436	1057 (73.6%)	379 (26.4%)	1.21 (1.03-1.43)	0.02
-	NA	1832	816 (44.5%)	1016 (55.5%)	1.45 (1.25-1.69)	<0.05
Stage	I	3228	2049 (63.5%)	1179 (36.5%)	Ref	-
-	II	951	520 (54.7%)	431 (45.3%)	1.63 (1.46-1.82)	＜0.05
Chemotherapy	Yes	3005	1909 (63.5%)	1096 (36.5%)	Ref	-
-	No	1174	660 (56.2%)	514 (43.8%)	1.18 (1.06-1.31)	<0.05

**Table 5:** Univariate analysis of stage III-IV OCCC and HGSC patients in the SEER database.

**Variables**		**No. of Patients**	**Survival (n)**	**Death (n)**	**HR (95%CI)**	***P-*Value**
Pathology type	OCCC	1141	281 (24.6%)	860 (75.4%)	Ref	**-**
-	HGSC	8639	1380 (16.0%)	7259 (84.0%)	0.81(0.75-0.87)	<0.05
Age(yr)	<60	4075	897 (22.0%)	3178 (78.0%)	Ref	-
-	≥60	5705	764 (13.4%)	4941 (86.6%)	1.38(1.32-1.45)	<0.05
Race	White	8155	1293 (15.9%)	6862 (84.1%)	Ref	-
-	Others	1625	368 (22.6%)	1257 (77.4%)	0.96 (0.91-1.03)	0.27
Laterality	Unilateral	3730	800 (21.4%)	2930 (78.6%)	Ref	-
-	Bilateral	6050	861 (14.2%)	5189 (85.8%)	1.18(1.13-1.23)	＜0.05
Size(mm)	<87	2159	523 (24.2%)	1636 (75.8%)	Ref	-
-	≥87	1801	569 (31.6%)	1232 (68.4%)	0.85(0.79-0.92)	<0.05
-	NA	5820	569 (9.8%)	5251 (90.2%)	1.18(1.12-1.25)	<0.05
Stage	III	6413	1242 (19.4%)	5171 (80.6%)	Ref	-
-	IV	3367	419 (12.4%)	2948 (87.6%)	1.40(1.34-1.47)	<0.05
Chemotherapy	Yes	8188	1447 (17.7%)	6741 (82.3%)	Ref	-
-	No	1592	214 (13.4%)	1378 (86.6%)	1.42(1.34-1.50)	<0.05

**Table 6:** Univariate analysis of prognostic factors for OCCC patients in SEER database.

**Variables**		**No. of Patients**	**Survival (n)**	**Death (n)**	**HR (95%CI)**	***P-*Value**
Age(yr)	＜60	2336	1470(62.9%)	866(37.1%)	Ref	-
-	≥60	1357	616(45.4%)	741(54.6%)	1.78(1.61-1.96)	＜0.05
Race	White	2682	1468(54.7%)	1214(45.3%)	Ref	-
-	Others	1011	618(61.1%)	393(38.9%)	0.90(0.80-1.01)	0.06
Laterality	Unilateral	3158	1960(62.1%)	1198(62.9%)	Ref	-
-	Bilateral	535	126(23.6%)	409(74.6%)	3.55(3.17-3.98)	＜0.05
Size(mm)	＜87	688	488(70.9%)	200(29.1%)	Ref	-
-	≥87	1470	954(64.9%)	516(35.1%)	1.31(1.11-1.54)	＜0.05
-	NA	1535	644(42.0%)	891(58.0%)	1.50(1.28-1.75)	＜0.05
Stage	I-II	2552	1805(70.7%)	747(29.3%)	Ref	-
-	III-IV	1141	281(24.6%)	860(75.4%)	5.41(4.89-5.98)	＜0.05
Chemotherapy	Yes	2769	1571(56.7%)	1198(43.3%)	Ref	-
-	No	924	515(55.7%)	409(44.3%)	0.95(0.85-1.06)	0.34

**Table 7:** Univariate analysis of prognostic factors for HGSC patients in SEER database.

**Variables**		**No. of Patients**	**Survival (n)**	**Death (n)**	**HR (95%CI)**	***P-*Value**
Age(yr)	＜60	4143	1170(28.2%)	2973(71.8%)	Ref	-
-	≥60	6123	974(15.9%)	5149(84.1%)	1.56(1.50-1.64)	＜0.05
Race	White	8680	1732(20.0%)	6948(80.0%)	Ref	-
-	Others	1586	412(26.0%)	1174(74.0%)	0.94(0.88-1.00)	0.06
Laterality	Unilateral	4087	1105(27.0%)	2982(73.0%)	Ref	-
-	Bilateral	6179	1039(16.8%)	5140(83.2%)	1.38(1.32-1.44)	＜0.05
Size(mm)	＜87	2382	731(30.7%)	1651(69.3%)	Ref	-
-	≥87	1767	672(38.0%)	1095(62.0%)	0.79(0.73-0.85)	＜0.05
-	NA	6117	741(12.1%)	5376(87.9%)	1.23(1.16-1.30)	＜0.05
Stage	I-II	1627	764(47.0%)	863(53.0%)	Ref	-
-	III-IV	8639	1380(16.0%)	7259(84.0%)	2.77(2.58-2.98)	＜0.05
Chemotherapy	Yes	8424	1785(21.2%)	6639(78.8%)	Ref	-
-	No	1842	359(19.5%)	1483(80.5%)	1.20(1.31-1.27)	＜0.05

**Table 8:** Clinical characteristics of 15 OCCC and 16 HGSC patients.

**Variables**		**Pathology Type**		**t/χ^2^**	***P-*Value**
		**OCCC (n = 15)**	**HGSC (n = 16)**	**-**	
Age(yr)		49.5±6.9	53.1±9.4	-1.2	0.23
Laterality	Unilateral	13	9	-	0.30
-	Bilateral	2	7	-	-
Size(mm)		13.5±4.2	11.5±4.6	1.24	0.25
Size(mm)	＜87	2	6	-	0.22
-	≥87	13	10	-	-
Stage	I-II	8	8	0.03	0.85
-	III-IV	7	8	-	-
Surgery	Yes	15	16	-	-
-	No	0	0	-	-
Chemotherapy	Yes	14	16	-	0.48
-	No	1	0	-	-
Status	Survival	12	14	-	0.65
-	Death	3	2	-	-

**Table 9:** Somatic gene mutation SNV and InDel location distribution information.

**ID Stage**		**Distribution of Somatic Gene Mutation SNV Location**					**Distribution of Somatic Gene Mutation InDel Locations**				**TMB**	
		**Missense Variant**	**Start Lost**	**Stop Gained**	**Stop Lost**	**Synonymous Variant**	**Frameshift Variant**	**Start Lost**	**Stop Gained**	**Stop Lost**	**Total**	**Mut/Mb**
A1	I	16981	51	993	17	9840	186	4	3	2	28502	570.04
A2	I	35603	95	2350	58	19671	125	2	5	2	17621	352.42
A3	I	23238	57	1482	31	12459	66	3	2	2	19239	384.78
A4	I	31044	72	1878	47	17095	113	1	16	0	23960	479.2
A5	I	2198	6	125	1	1259	84	4	3	2	4872	97.44
A6	I	16722	52	1078	27	9361	136	2	5	3	31779	635.58
A7	II	10222	37	714	11	5767	151	1	5	1	35122	702.44
A8	II	3608	9	214	4	2159	136	4	2	0	2401	48.02
A9	III	27760	95	1889	31	14454	44	1	1	1	17305	346.1
A10	III	18521	52	1268	15	10669	159	3	7	4	36793	735.86
A11	III	4512	13	293	7	2674	102	5	1	2	10802	216.04
A12	III	10268	40	670	16	6077	93	2	3	7	3998	79.96
A13	III	11672	27	786	14	6788	80	1	4	1	2224	44.48
A14	III	34192	73	2279	44	18192	60	0	11	1	10727	214.54
A15	III	2096	5	119	3	1188	26	1	1	2	12110	242.2
B1	II	45631	123	3118	56	25877	75	2	0	1	46979	939.58
B2	II	17835	53	1188	29	10036	126	2	5	0	11987	239.74
B3	I	11344	32	749	15	6658	160	3	5	4	30146	602.92
B4	I	28699	74	1883	54	15599	60	2	1	0	29479	589.58
B5	I	4410	8	266	4	2499	70	2	0	2	5619	112.38
B6	II	30428	65	2093	46	17142	78	1	1	0	24856	497.12
B7	II	19047	43	1277	26	10614	133	4	2	1	4606	92.12
B8	I	2641	6	131	2	1502	102	4	4	1	4580	91.6
B9	IV	29404	69	1923	37	15839	51	1	1	1	31413	628.26
B10	III	5272	9	338	6	3113	110	3	0	4	5292	105.84
B11	III	24176	65	1547	45	13424	61	4	1	4	19792	395.84
B12	III	4390	12	229	2	2571	44	1	0	1	36211	724.22
B13	III	4942	9	312	4	2880	119	1	0	1	2844	56.88
B14	III	35207	71	2347	42	19349	48	0	1	1	18528	370.56
B15	III	4797	7	308	2	2700	78	4	1	1	4363	87.26
B16	IV	4017	10	259	2	2429	97	2	0	2	5089	101.78

**Table 10:** The top 20 SMGs in stage III-IV OCCC and stage III-IV HGSC uncover novel drug targets from the DGIdb database.

**Genes**	**Drugs**	**Regulatory Approval**	**Indications**	**Interaction Scores**
NOTCH1	Hydrocortisone butyrate	Approved	NA	0.31
	Ro4929097	Not approved	NA	3.92
	Crenigacestat	Not approved	NA	2.18
	Mk-0752	Not approved	NA	1.31
	Brontictuzumab	Not approved	NA	19.58
	Nirogacestat	Approved	NA	1.40
	Enoticumab	Not approved	NA	1.31
SYNE2	Vincristine	Approved	Antineoplastic agent	1.29
LAMA5	Ocriplasmin	Approved	-	1.37
	Amlodipine besylate	Approved	Cardiovascular and antihypertensive agent	0.87
	Adenosine triphosphate	Approved	NA	0.38
	Ryanodine	Not approved	NA	1.63
	Dantrolene	Approved	Muscle Relaxants	1.45
	Ruthenium red	Not approved	NA	0.82
RYR3	Ca2^+^	Not approved	NA	0.16
	Mg2^+^	Not approved	NA	0.50
	Caffeine	Approved	Central Nervous System Stimulants	0.18
MUC5AC	Yttrium Y90 clivatuzumab tetraxetan	Not approved	NA	13.05
-	Ensituximab	Not approved	NA	6.53
-	Clivatuzumab tetraxetan	Not approved	NA	13.05
-	Therapeutic glucocorticoid	Not approved	NA	0.77
HERC2	Neratinib	Approved	Antineoplastic agent	6.96
MUC16	Oregovomab	Not approved	Antineoplastic agent	22.37
	Topotecan hydrochloride	Approved	Antineoplastic agent	0.31
	Sodium butyrate	Not approved	NA	0.80
	Igovomab	Approved	NA	3.73
	Differentiation inducer	Not approved	NA	0.68
	Docetaxel anhydrous	Approved	Antineoplastic agent	0.13
	Sofituzumab vedotin	Not approved	NA	0.23
	Tamoxifen	Approved	Hormonal, Antineoplastic agents	0.11
	Buserelin acetate	Approved	NA	7.46
	Etoposide	Approved	Antineoplastic agents	0.13
	Cyclosporine	Approved	Immunosuppressant and opthalmological agent	0.09
	Abagovomab	Not approved	Antineoplastic agent	11.19
	Recombinant interferon	Not approved	NA	0.19
	Sofituzumab vedotin	Not approved	NA	3.73
RYR1	Simvastatin	Approved	Antidyslipidaemic, anticholesterolaemic, and antihypertensive agent	0.15
	Halothane	Approved	Inhalation anesthetics	13.36
	Desflurane	Approved	Inhalation anesthetics	16.03
	Methoxyflurane	Approved	Inhalation anesthetics	21.70
	Dantrolene	Approved	Muscle relaxants	2.07
	Succinylcholine	Approved	Skeletal and muscle relaxants	51.53
	Arm-210	Not approved	NA	3.73
	Calcium	Approved	NA	3.73
	Haloperidol decanoate	Approved	Antipsychotic agents	0.10
	Rocuronium	Approved	Muscle relaxant	1.07
	Enflurane	Approved	Inhalation anesthetics	15.40
	Ryanodine	Not approved	NA	1.86
	Sevoflurane	Approved	Inhalation anesthetics	0.10
	Atorvastatin calcium trihydrate	Approved	Anticholesterolaemic and antihypecholesterolemic agent	0.14
HSPG2	Cyclosporine	Approved	Immunosuppressant and opthalmological agent	0.40
	Haloperidol decanoate	Approved	Antipsychotic agents	0.46
	Palifermin	Approved	For the treatment of antineoplasty-induced oral mucositis	2.18

## Data Availability

All the data and supporting information are provided within the article.

## LIST OF ABBREVIATIONS

95%CI: 95% Confidence Interval
ARID1A: AT-rich Interaction Domain 1A
ARID3A: AT-rich Interaction Domain 3A
AJCC: American Joint Committee on Cancer
BRCA1/2: Breast Cancer Susceptibility gene 1/2
BARD1: BRCA1 associated RING domain 1
CTAB: Cetyltrimethylammonium Bromide
cGMP-PKG: Cyclic Guanosine Monophosphate-Protein Kinase
DNAH2: Dynein Axonemal Heavy Chain 2
EOC: Epithelial Ovarian Carcinoma
ECM: Extracellular Matrix
FFPE: Formalin-Fixed Paraffin-Embedded
FIGO: International Federation of Gynecology and Obstetrics
FCPT: Fisher's Combined P-value Test
HDAC: Histone Deacetylase
HGSC: High-Grade Serous Carcinoma
HR: Hazard Ratio
HIF-1: Hypoxia Inducible Factor-1
InDel: Insertion-Deletion
KEGG: Kyoto Encyclopedia of Genes and Genomes
LAMA5: Laminin Subunit Alpha 5
LRT: Likelihood Ratio Test
LGSC: Low-Grade Serous Carcinoma
Mut/Mb: Total Number of Mutations per Megabase
MUC4: Mucin 4
MUC19: Mucin 19
MAGEE1: MAGE Family Member E1
MMR: Mismatch Repair
NA: Not Available
ns: Not Significant
NOTCH1: Neurogenic Locus Notch Homolog Protein 1
OCCC: Ovarian Clear Cell Carcinoma
OS: Overall Survival
PCLO: Piccolo Presynaptic Cytomatrix Protein
PIK3CA: Phosphatidylinositol-4,5-Bisphosphate 3-Kinase Catalytic Subunit Alpha
PFS: Progression-Free Survival
RCTs: Randomized Controlled Trials
SEER: Surveillance, Epidemiology, and End Results
SNV: single-nucleotide Variation
SMGs: Significantly Mutated Genes
SWI/SNF: SWItch/Sucrose Non-Fermentable
SYNE2: Spectrin Repeat Containing Nuclear Envelope Protein 2
TMB: Tumor Mutation BurdenC
TACC2: Transforming Acidic Coiled-Coil Containing Protein 2
WES: Whole Exome Sequencing
ZNF469: Zinc Finger Protein 469

## STANDARDS OF REPORTING

STROBE guidelines were followed.
